# Effects of post-scenario debriefing versus stop-and-go debriefing in medical simulation training on skill acquisition and learning experience: a randomized controlled trial

**DOI:** 10.1186/s12909-019-1772-y

**Published:** 2019-09-05

**Authors:** Patrick Schober, Kay R. J. Kistemaker, Fereshte Sijani, Lothar A. Schwarte, Dick van Groeningen, Ralf Krage

**Affiliations:** 1 Department of Anesthesiology, Amsterdam UMC, Vrije Universiteit Amsterdam, De Boelelaan 1117, 1081HV Amsterdam, the Netherlands; 2 Department of Cardiology, Noordwest Ziekenhuisgroep, location Alkmaar, Alkmaar, the Netherlands; 3 Medical Student, Vrije Universiteit Amsterdam, Amsterdam, the Netherlands

**Keywords:** Medical education, Debriefing, Simulation

## Abstract

**Background:**

Debriefing is a critical component to promote effective learning during simulation-based training. Traditionally, debriefing is provided only after the end of a scenario. A possible alternative is to debrief specific portions during an ongoing simulation session (stop-and-go debriefing). While this alternative has theoretical advantages, it is not commonly used due to concerns that interruptions disturb the fidelity and adversely affect learning. However, both approaches have not been rigorously compared, and effects on skill acquisition and learning experience are unknown.

**Methods:**

We randomly assigned 50 medical students participating in a simulation-based cardiopulmonary resuscitation training to either a post-scenario debriefing or stop-and-go debriefing. After four weeks, participants performed a repeat scenario, and their performance was assessed using a generic performance score (primary outcome). A difference of 3 or more points was considered meaningful. A 5-item questionnaire was used to assess the subjective learning experience and the perceived stress level (secondary outcomes).

**Results:**

There was no significant difference between the groups for the performance score (mean difference: -0.35, 95%CI: -2.46 to 1.77, *P* = 0.748, *n* = 48). The confidence limits excluding the specified relevant 3-point difference suggest equivalence of both techniques with respect to the primary outcome. No significant differences were observed for secondary outcomes.

**Conclusions:**

Stop-and-go debriefing does not adversely affect skill acquisition compared to the classic post-scenario debriefing strategy. This finding is reassuring when interruptions are deemed necessary and gives simulation instructors the latitude to tailor the timing of the debriefing individually, rather than adhering to the unsupported dogma that scenarios should not be interrupted.

**Trial registration:**

As this study is not a clinical trial, it was not registered in a clinical trials register.

## Background

Simulation-based learning is widely used in the education of medical personnel to train skills, knowledge, and teamwork in a safe environment [1]. Herein, debriefing is considered a critical component to promote effective learning [2, 3].

Traditionally, a debriefing session is provided only after the end of a scenario. A possible alternative could be to time-out the scenario to debrief specific portions or events during an ongoing simulation session. There are two potential advantages of this in-scenario or stop-and-go approach. One, the participants might be better able to recall the events during the debrief sessions when the simulation is paused immediately after the event. Two, the repetitive short debriefings may be more easily “digestible” and could lead to an enhanced acquisition of skills and knowledge than one extensive debriefing at the end of the session. Moreover, errors can be corrected in a more timely manner before they occur repeatedly and consolidate during a scenario. The disadvantage of interrupting a scenario is an inherent reduction in the level of fidelity, and it might affect the participant’s engagement in the scenario [4].

Differences between stop-and-go debriefing and post-scenario debriefing have not been rigorously studied, and the net effects of the potential advantages and disadvantages on the learning experience are largely unknown. To our knowledge, only one study previously compared these debriefing approaches [5]. Based on the questionnaire responses, these authors reported that post-scenario debriefing was more effective compared to debriefing that occurred during the scenario. However, the authors only studied self-reported outcomes, but did not objectively measure skill or knowledge acquisition. Moreover, it was not only the timing, but also the type of debriefing that was different between the groups. Participants in the in-scenario group were instructed on what they should do whenever there was an error or when an action was omitted, whereas the participants of the post-scenario group received a debriefing that stimulated a reflection of their actions and included supportive encouragement. Hence, the results of this study are likely confounded by the type of debriefing and provide limited information on differences between stop-and-go versus post-scenario debriefing.

We therefore aimed to determine whether one of the approaches results in better skill acquisition after the training of a cardiopulmonary arrest scenario and tested the null hypothesis of no difference between the groups. As a secondary aim, we assessed the subjective perception of the participants’ learning experience.

## Method

This study was performed at the clinical ADAM (“Anesthesiology Directed to Acute Medicine”) simulation center of the Amsterdam University Medical Center, Location VUmc, in Amsterdam, the Netherlands. A written informed consent was obtained from all the participants. This study did not involve patients and did not subject volunteers to any conditions other than a standard training situation and does not fall under the Medical Research Involving Human Subjects Act (WMO). Therefore, formal approval of the Institutional Review Board was not required by Dutch law. As this investigation is not a clinical trial, it was not registered in a clinical trials register.

### Participants

Fifty medical students who had previously attended basic life support (BLS) training were recruited to participate in a scenario-based cardiopulmonary resuscitation training using a patient simulator (ALS simulator, Laerdal Medical Corporation, Stavanger, Norway). Participants were informed that the study involved two scenarios: one scenario served as a training scenario and involved debriefing, and the second without debriefing served to assess their performance. Both scenarios involved simulations separated by a four-week period. All participants were oriented to the patient simulator and the simulation environment, including the available automatic external defibrillator (AED), before the initiation of the study. They also watched a BLS instruction video provided by the European Resuscitation Council (ERC) as a refresher, to ensure a uniform level of theoretical knowledge at the beginning of the study.

Thereafter, students were randomly assigned to the either of the two possible debriefing techniques using a sealed envelope technique. While the students were aware that different teaching techniques would be compared, they were not aware of the specific differences between the approaches and did not know to which group they had been assigned.

### Clinical simulation scenario

The scenario included a patient with a history of unstable angina pectoris, who is found unresponsive on the ward by a nurse (played by a member of the research team). The participant was unaware that the patient was in cardiac arrest. The nurse called the participant for immediate assistance, who was then expected to assess the patient, detect absence of circulation, and to initiate cardiopulmonary resuscitation (including the use of the AED) according to current resuscitation guidelines [6]. The nurse was instructed to provide assistance with necessary measures on the student’s request (e.g., to call the resuscitation team, retrieve and attach the AED, or take over chest compressions). At one minute after the AED’s second rhythm analysis, the resuscitation team arrived. The scenario ended with a handover of the patient from the participant to the resuscitation team.

### Debriefing

The participants were debriefed after or during the training scenario by one of the two experienced instructors of our simulation center (RK or PS). Both have several years of experience using different debriefing and feedback techniques. For this study, the learning conversation was used [7, 8]. This technique is a learner-centered dialogue that stimulates reflection by allowing the learner to identify and explore issues of particular interest, and in which the instructor uses the *‘advocacy with inquiry’* technique to share his or her thoughts on learning points that have not been raised by the learner [9].

According to the group allocation, participants were debriefed either after the end of the scenario (post-scenario group), or during the scenario (stop-and-go group) at three prespecified time points:

1. Before the initiation of the hands-on cardiopulmonary resuscitation (BLS): This debriefing addressed the initial patient assessment and the proper recognition of cardiac arrest, the need to notify the resuscitation team and to retrieve a defibrillator, and non-technical aspects such as team communication and task allocation.
2. After the initial defibrillation with the AED: Participants were debriefed to discuss the technical aspects of chest compressions and ventilations, as well as the proper and safe use of the defibrillator.
3. After the end of the scenario: Participants were debriefed to reinforce technical and non-technical aspects of the resuscitation, to discuss differences in the treatment of a shockable versus non-shockable heart rhythm, and to review communication strategies to effectively hand over a patient to the resuscitation team.

The targeted overall time for the debriefings was comparable in both groups (approximately 15 min post-scenario versus 3 times 5 min during stop-and go). The instructor was present in the simulation room in both groups.

### Outcome measures

A summative assessment of the participants’ performance was conducted using a second scenario at four weeks after the initial training scenario. The scenario was identical to the first, with the exception that the instructors were not present in the simulation room and that participants were not debriefed. As no validated instrument exists to assess performance during BLS, we developed a scoring protocol to summarize the participants’ performance for the purpose of this study, based on the experience with previous studies of our research group in which a similar scoring protocol was developed [10, 11]. The present score ranges from − 28 to + 32 points and represents the weighted sum of a number of elements that are considered crucial for a safe and efficient resuscitation [6]. Based on a scoring sheet, points were added for appropriate actions (e.g., activation of the resuscitation team on the detection of cardiac arrest: + 1 point) or subtracted for inappropriate actions. Inappropriate actions were the omission of indicated actions (e.g., omission of AED use: − 4 points), inappropriate execution of indicated actions (e.g., chest compressions to shallow or outside the target rate, points assigned depending on number, depth and frequency), or time delays (e.g., delays in initiating chest compressions, points assigned depending on the delay). Two members of the research group (KK, FS) reviewed video recordings and scored the participant’s performance. As the aim of the study was not the validation of the score but rather to obtain a score that most accurately reflected the participant’s performance, the raters discussed any disagreements and videos were re-reviewed until a consensus on the participant’s performance was reached. A difference in the performance scores between the groups was the primary outcome of this study.

The subjective learning experience (secondary outcome) was assessed by a questionnaire distributed to the participants immediately after the training session. The questionnaire was developed by the research team for the purpose of this study and consisted of four Likert-type questions, with answer options ranging from 1 (completely disagree) to 5 (completely agree). A fifth question asked about the perceived stress level during the scenario, ranging from 1 (absolutely no stress) to 5 (maximum stress level).

### Statistical analysis

The study was powered to detect a 3-point difference of the performance score between the groups with 90% power at a 0.05 alpha level and assuming a common standard deviation of 3. A 3-point difference or larger was considered clinically relevant as it corresponds to approximately a 5% difference across the range of possible scores. The calculated sample size of 46 (23 per study arm) was inflated to 50 to account for possible dropouts.

Data were analyzed with STATA 15.1 (StataCorp, College Station, TX, USA). To adjust for possible differences between the two instructors who conducted the debriefings, performance scores were compared between the groups using linear regression, in which the group allocation and debriefer were the binary independent variables and the performance score was the dependent variable. As a Q-Q plot suggested a non-normal distribution of the performance score, standard errors were bootstrapped using 10,000 replications. A group-by-instructor interaction was considered, but was dropped from the model, as it was not significant. For a sensitivity analysis, the aforementioned model was additionally adjusted for baseline imbalances (absolute standardized differences > 0.1, Table 1). A non-parametric group comparison using the Mann-Whitney U test served as a second sensitivity analysis, and the shift in the location parameter was estimated with the Hodges-Lehmann estimator. Ordinal outcomes (Likert scale items) were compared using the Mann-Whitney U test. Two-sided *P*-values < 0.05 were considered significant.

**Table 1:** Baseline characteristics of participants in both groups

Variable	Post-scenario group *n* = 24	Stop-and-Go group *n* = 24	Standardized Difference
Sex (male/female)	6/18 (25%/75%)	10/14 (42%/58%)	0.359
Age (years)	21 [20; 22 (19–26)]	21 [20; 23 (19–32)]	0.177
Year of study	2.5 [2; 4 (1–6)]	2 [2; 4 (1–5)]	0.097

Data are presented as number (percentage) or median [quartiles (range)]

## Results

Of the 50 participants, two were excluded (one in each group) due to technical problems with the video recording, since their performance scores could not be assessed. The participants were mostly female (*n* = 33/48, 69%) had a median (quartiles) age of 21 (20; 22) years and were in their second (2nd; 4th) study year (Table 1).

No statistically significant difference was found between the groups for the performance score in the main analysis (post-scenario debriefing: 24.10; stop-and-go debriefing: 23.75; mean difference: − 0.35, 95% confidence interval: − 2.46 to 1.77, *P* = 0.75, model R^2^ = 0.003). The confidence limits did not cross the specified clinically relevant 3-point difference mark in any direction, suggesting a clinical equivalence of both debriefing techniques with respect to the primary outcome. Sensitivity analyses showed consistent results (*P* = 0.97 and *P* = 0.95, respectively, with 95% confidence intervals of mean difference and location shift within − 3 to + 3).

With respect to the secondary outcomes, there were no significant differences between the groups for any questionnaire items of personal learning experience or perceived stress level (Table 2).

**Table 2:** Secondary outcomes

	Post-scenario group *n* = 24	Stop-and-Go group *n* = 24	*P*-value
Question 1: *“I had the feeling that the feedback I received was a useful contribution to my learning experience.”*			
Median [quartiles (range)]	5 [5; 5 (4–5)]	5 [5; 5 (4–5)]	1.00
Question 2: *“I felt comfortable with the way that I received feedback.”*			
Median [quartiles (range)]	5 [5; 5 (4–5)]	5 [5; 5 (4–5)]	0.08
Question 3: *“The feedback over my strengths was clear to me.”*			
Median [quartiles (range)]	5 [4; 5 (3–5)]	5 [4; 5 (4–5)]	0.29
Question 4: *“The feedback over my points of improvement was clear to me.”*			
Median [quartiles (range)]	5 [5; 5 (4–5)]	5 [5; 5 (4–5)]	0.23
Question 5: *“My stress level during the scenario was:”*			
Median [quartiles (range)]	3 [3; 4 (2–4)]	3 [2; 3.5 (1–4)]	0.21

Questionnaire items assessed by Likert-type scale scores ranging from 1 = completely disagree to 5 = completely agree. For stress, 1 means that the participant experienced absolutely no stress and 5 represents maximum stress

## Discussion

In this study, we compared the skill acquisition and learning experience after post-scenario debriefing versus stop-and-go debriefing in medical students and found no evidence for a difference between both approaches.

A variety of debriefing and feedback approaches have been described in literature, including the Pendleton technique [7], the sandwich method [8], the diamond structure [12], the blended PEARLS approach [13], and the learning conversation [7–9]. Debriefing can be instructor-led or peer-led [14], can be video-assisted or not [15], and can focus on different aspects of performance such as technical skills or nontechnical performance, including task management, situational awareness, leadership, communication, and decision-making. An extensive discussion of the differences and relative advantages of each technique is beyond the scope of this manuscript. However, it is important to realize that the differences refer to the manner in which the debriefing is provided, not to the timing of the debriefing. While a number of studies have compared different debriefing techniques [16, 17], literature on different approaches for timing of the debriefing is virtually lacking. Yet, the timing could substantially influence the learning experience and knowledge acquisition, and therefore warrants an investigation.

Although a debriefing is usually conducted at the end of a scenario, it may be necessary to suspend a scenario when the performance is seriously flawed [2]. Independent of the debriefing technique, errors can then be corrected early, before they are repeated continually. Routine interruptions to debrief portions of the scenario could also be useful, as participants may better recall the events being debriefed, and acquisition of learning points might theoretically be improved when ingested in smaller quantities. Interruptions of a scenario potentially also have negative effects, including a reduction in fidelity and altered engagement of the participant in the scenario [4].

In this context, a novel learning approach termed “Rapid Cycle Deliberate Practice” (RCDB) has increasingly been advocated in the last few years, particularly in the fields of pediatric and neonatal resuscitation [18–20]. This approach focuses on mastery learning in which the learners cycle back and forth between deliberate skill practice and brief corrective feedback until a skill is mastered. Data on the effects of this approach on skill acquisition are yet scarce, and this repetitive alternation between directed corrective feedback and skill practice is clearly different from the reflective feedback techniques traditionally used in medical simulation debriefing [21, 22]. Nonetheless, this approach and stop-and-go debriefing have in common a skill practice or scenario that is interrupted, and promising initial experiences with RCDB underline the potential usefulness of the repetitive interruptions of a scenario in a stop-and-go debriefing approach.

In this present study, we did not observe any significant differences between both debriefing techniques with respect to skill acquisition, learning experience or perceived stress. This study was not designed as an equivalence trial. However, the narrow confidence interval around the mean difference does not include clinically important differences, suggesting that the choice of stop-and-go versus post-scenario debriefing has no relevant effect on skill acquisition at 4 weeks after the training [23]. Similarly, the R^2^-value of the regression model of virtually zero suggests that the timing of debriefing explains none of the observed variability of the performance scores [24, 25]. In addition, no evidence for differences of any secondary outcomes was observed.

Oriot and Alinier suggest that in-scenario debriefing is not appropriate for scenario-based learning [4], and from our experience, instructors often view it as a dogma not to interrupt a scenario unless strictly necessary. In contrast, our results suggest that both approaches can be used interchangeably with equivalent effects on learning. We think that this finding is an important discovery because it gives instructors more latitude to decide how, and especially when, to engage participants in a learning discussion and allows instructors to tailor the debriefing individually to the scenario and its participants. Moreover, the results of our study give instructors the freedom to alternate post-scenario and stop-and-go debriefing when several scenarios are played in sequence. Previously, Carr advocated the use of a variety of different feedback techniques to avoid that the experience becomes predictable [26]. We speculate that diversification and alternation not only in the debriefing techniques, but also in their timing, may increase the participant’s attention and enhance the simulation learning experience. This diversification and alternation, in turn, may favorably contribute to the learning experience.

This study has several limitations. First, we used the learning conversation as a debriefing technique, and the results cannot necessarily be extrapolated to other techniques. Second, the study was performed with medical students as participants, and results do not necessarily apply to other participant groups. Third, no validated instrument was available to score the participants’ performance during the second scenario. Available checklists to measure BLS performance, such as the High Quality BLS Skills Testing Checklist by the American Heart Association, have binary checkboxes but do not provide a score and do not allow assessing the performance on a continuous scale. For example, chest compressions with a frequency of 95/min are below the target range, but are clearly better than a compression frequency of 70/min or not performing any chest compressions at all. However, in all of these cases, the respective check box would not be checked thereby precluding the ability to differentiate between different gradations of performance. Further, checkboxes weight all items equally, irrespective of their relative importance. We therefore created a scoring protocol from scratch that provides a continuous score, based on current resuscitation guidelines and based on the prior experience of our research groups with similar scoring systems. While the score has face validity, a complete formal validation and assessment of psychometric properties including reliability and criterion validity has not yet been performed. However, the scoring technique was used in both groups. We believe that the between-group’s score difference should give a valid assessment of performance differences between in-scenario versus post-scenario debriefing. Fourth, differences in skills were only assessed at 4 weeks after the initial training scenario, such that the data does not allow conclusions on long-term skill retention. Finally, we did not measure the baseline performance of the participants. Therefore, we can only draw conclusions on the differences between the groups in the second scenario. Future studies should examine how different debriefing techniques affect performance over time.

## Conclusions

Stop-and-go debriefing does not adversely affect skill acquisition or subjective learning experiences as compared to the classic post-scenario debriefing strategy. This finding should encourage simulation instructors to tailor the timing of the debriefing to the specific needs of the participants rather than adhering to a protocol.

## Data Availability

The dataset used and analyzed during the current study are available from the corresponding author on reasonable request.

## Abbreviations

AED: Automatic External Defibrillator
ALS: Advanced Life Support
BLS: Basic Life Support
ERC: European Resuscitation Council.
VUmc: VU University Medical Center
WMO: Wet medisch-wetenschappelijk onderzoek met mensen (Medical Research Involving Human Subjects Act)
